# Multiple polymorphisms, but no mutations, in the WAF1/CIP1 gene in human brain tumours.

**DOI:** 10.1038/bjc.1995.491

**Published:** 1995-11

**Authors:** J. Koopmann, D. Maintz, S. Schild, J. Schramm, D. N. Louis, O. D. Wiestler, A. von Deimling

**Affiliations:** Institut für Neuropathologie, Universitätskliniken Bonn, Germany.

## Abstract

**Images:**


British Journal of Cancer (1995) 72, 1230–1233

© 1995 Stockton Press All rights reserved 0007–0920/95 $12.00

### Multiple polymorphisms, but no mutations, in the WAF1/CIP1 gene in human brain tumours

J Koopmann[1], D Maintz[1], S Schild[1], J Schramm[2], DN Louis[3], OD Wiestler[1] and A von Deimling[1]

[1]*Institut für Neuropathologie, Universitätskliniken Bonn, Germany;* [2]*Neurochirurgische Klinik, Universitätskliniken Bonn, Germany;* [3]*Molecular Neuro-Oncology Laboratory, Neurosurgical Service and Department of Pathology (Neuropathology), Massachusetts General Hospital, Boston, MA, USA.*

**Summary** The cyclin kinase inhibitor WAF1/CIP1, also termed CDKN1, mediates p53-induced cell cycle arrest in response to DNA damage. This property makes it an attractive tumour-suppressor candidate for a p53-associated tumour-suppressor gene. In order to investigate the role of WAF1/CIP1 in the pathogenesis of primary human brain tumours we performed single-stranded conformation polymorphism (SSCP) analysis and direct sequencing of exon 2 of the gene in a representative series of 158 brain tumours and corresponding blood samples. In addition, all tumours were examined for mutations in exons 5–8 of the p53 gene. Analysis of WAF1/CIP1 revealed multiple polymorphisms, the most abundant being AGC→AGA (Ser→Arg) at codon 31 with an allele frequency of 8.5%. Less common polymorphisms included GTG→GGG (Val→Gly) at codon 25, GCC→ACC (Ala→Thr) at codon 64, CGC→CTC (Arg→Leu) at codon 32, GGC→AGC (Gly→Ser) at codon 14 and GCG→GTG (Ala→Val) at codon 39 each with an allele frequency of 0.3%. These polymorphisms were all located in a conserved region of exon 2. Two of the polymorphisms were also seen in a group of 157 healthy controls indicating that WAF1/CIP1 polymorphisms do not predispose to cancer. None of the tumours included in our series showed a somatic mutation in WAF1/CIP1. All samples were also analysed for loss of heterozygosity on the short arm of chromosome 6 in the region of the WAF1/CIP1 locus. Allelic loss was observed in only one patient with a glioblastoma. Mutations in the p53 gene were found in 22 of 158 tumours. No association was found between any polymorphism of the WAF1/CIP1 gene, p53 mutations and histopathological tumour type. Our data indicate that WAF1/CIP1 mutations are probably not involved in the formation of primary human brain tumours.

**Keywords:** WAF1; CIP1; p21; p53; CDKN1; brain tumours

The p53 tumour-suppressor gene is frequently mutated in human brain tumours (Louis, 1994). Wild-type p53 plays a major role in the cellular response to DNA damage, either by inducing a $G_1$ arrest and subsequent DNA repair or by inducing apoptosis (Lane, 1993). Some of these effects are likely to be executed by genes which act as downstream mediators of p53 (Smith *et al.*, 1994). Recently a link between p53 and cell cycle arrest was provided by the cloning of the cyclin kinase inhibitor WAF1/CIP1, also called CDKN1 or $p21^{CKI}$ (El-Deiry *et al.*, 1993; Harper *et al.*, 1993). WAF1/CIP1 transcription is directly induced by p53 in response to DNA damage. It blocks the $G_1$–S transition by inhibiting the activity of multiple cyclin–cyclin dependent kinase complexes (Xiong *et al.*, 1993; Di Leonardo *et al.*, 1994; El-Deiry *et al.*, 1994). Further, the WAF1/CIP1 gene product, p21, exerts a growth-suppressing effect if expressed under the control of a heterologous promoter (El-Deiry *et al.*, 1993).

This induction by p53 and its function as a negative regulator of the cell cycle make WAF1/CIP1 an attractive candidate for a tumour-suppressor gene. Alterations of WAF1/CIP1 function could constitute an alternative mechanism to p53 inactivation. Thus WAF1/CIP1 mutations may contribute to the development of those tumours who have retained wild-type p53 alleles. Several studies have demonstrated a high incidence of p53 mutations in human gliomas, but have also documented that many gliomas express wild-type p53 (von Deimling *et al.*, 1992; Rubio *et al.*, 1993; Louis, 1994). In order to evaluate the hypothesis that WAF1/CIP1 mutations interrupt the p53-mediated pathway in brain tumours with wild-type p53, we performed single-strand conformational polymorphism (SSCP) analysis of both the WAF1/CIP1 gene and the p53 gene in 158 human brain tumours. In addition we assessed these tumours for allelic loss in the WAF1/CIP1 region on chromosomal arm 6p.

Correspondence: A von Deimling, Institut für Neuropathologie, Universitätskliniken Bonn, Sigmund-Freud-Straße 25, D-53105 Bonn, Germany
Received 1 March 1995; revised 12 May 1995; accepted 19 May 1995

### Materials and methods

### *Tumour specimens, histopathology and control DNA*

Tumour and corresponding blood samples were obtained from patients at the Massachusetts General Hospital, Boston, the University Hospital, Zurich and the University Hospital, Bonn. All tumours were classified by a neuropathologist (AvD) according to the revised WHO guidelines (Kleihues *et al.*, 1993). The specimens were examined microscopically before phenolic DNA extraction (Sambrook *et al.*, 1989). The series of 158 tumours included 54 glioblastomas WHO grade IV, 15 anaplastic astrocytomas WHO grade III, nine astrocytomas WHO grade II, 22 pilocytic astrocytomas WHO grade I, 20 meningiomas WHO grade I, five atypical meningiomas WHO grade II, one anaplastic meningioma WHO grade III, 12 primitive neuroectodermal tumours WHO grade IV (including ten medulloblastomas), five oligodendrogliomas WHO grade II, two anaplastic oligodendrogliomas WHO grade III, six oligoastrocytomas WHO grade II, three anaplastic oligoastrocytomas WHO grade III and one each of schwannoma, neurofibroma, gangliocytoma and pleomorphic xanthoastrocytoma. Normal control DNAs were obtained from 157 unrelated healthy whites from Germany.

### *SSCP analysis*

For analysis of the WAF1/CIP1 gene primers for two overlapping portions of exon 2 were generated based on the published cDNA sequence (El-Deiry *et al.*, 1993). The amplified region constitutes 87% of the coding sequence of the WAF1/CIP1 gene. The primers were 5′-GTCAGAACC-GGCTGGGGATG and 5′-CTCCTCCCAACTCATCCCGG for fragment 1 (272 bp) and 5′-TGGCCTGCCCAAGCTC-

TACC and 5′-GGTCTGCCGCCGTTTTCGAC (223 bp) respectively. Polymerase chain reaction (PCR) was performed in a volume of 10 μl containing 10 ng of DNA, 50 mM potassium chloride, 10 mM Tris-HCl, 200 mM of each dNTP, 0.1% gelatin, 20 pmol of each primer, 1.0 mM magnesium chloride and 0.025 U of *Taq* polymerase. Initial denaturation at 94°C for 3 min was followed by 30 cycles on an automated thermal cycler (Hybaid, Omnigene, USA). These included denaturation at 94°C for 30 s, annealing at 57°C for 40 s and extension at 72°C for 40 s. A final extension step at 72°C for 10 min was added. SSCP was performed on a sequencing apparatus (Pokerface II, Hoefer, San Francisco, USA) using 12% acrylamide, run at 20 W, 14 h at 4°C for fragment 1, and 8% acrylamide run at 2 W, 14 h at 25°C for fragment 2. Silver staining of the gels was performed as previously described (von Deimling *et al.*, 1993; Bender *et al.*, 1994). Analysis of the p53 gene was carried out as reported elsewhere (von Deimling *et al.*, 1992).

*Direct sequencing*

Aberrantly migrating SSCP bands were excised and the DNA was extracted as described (Sambrook *et al.*, 1989). After reamplification with the same set of primers the PCR products were sequenced on a semiautomated sequencer (Applied Biosystems, model 373) using the corresponding *Taq* cycle sequencing kit. Each amplicon was sequenced bidirectionally.

*Microsatellite analysis for loss of heterozygosity*

The following primer pairs on chromosome 6p were used for a non-radioactive microsatellite analysis: D6S497 at 6p22.3–p21 and D6S105 at 6p22–p21.3. PCR products were separated on 8% denaturing acrylamide gels and silver stained. Loss of heterozygosity (LOH) was scored as previously described (Louis *et al.*, 1992).

### Results

SSCP analysis and direct sequencing of the WAF1/CIP1 gene were conducted with two primer pairs generating fragments of 272 bp and 223 bp respectively. These fragments encompass 87% of the coding sequence. None of these tumours exhibited a somatic mutation of the WAF1/CIP1 gene. However, we found six different polymorphisms which resulted in eight observed haplotypes (see Figure 1). These patterns were identical in both tumour and constitutional DNA of all patients with aberrant SSCP fragments. An overview of allele distribution and observed haplotypes is given in Tables I and II.

The study revealed six distinct polymorphisms designated A–F with two alleles each. Allele frequencies were 0.91/0.09 for A1/A2, and 0.997/0.003 for the polymorphisms B–F. The distribution of these polymorphisms yielded eight different haplotypes of which I and II were predominant. In this series haplotype I was detected in 80.4%, II in 15.8% and III to VIII in one patient each (0.63%).

In order to compare the rate of polymorphisms in the tumour group with the normal population we analysed a panel of DNAs from 157 healthy whites from Germany. Haplotype frequencies were I 80.2%, II 18.5% III and IV 0.64% (one patient each). The incidence of polymorphisms was similar in both tumour and control groups for A1/A2 and B1/B2. The polymorphisms C2, D2, E2 and F2 were only observed in the tumour group. Patients with the I and II haplotype shared a similar spectrum of histopathological tumour types and were not associated with a specific tumour entity.

Microsatellite analysis for LOH in the area of WAF1/CIP1 gene on 6p21.2 revealed a total of 106 informative cases in the brain tumour group. LOH was seen in only one patient with a glioblastoma.

Mutational analysis of exons 5–8 of the p53 gene in the primary brain tumours revealed 23 somatic sequence alterations in 22 tumours, corresponding to an overall mutation frequency of 13%. Mutations were seen in 11/54 glioblastomas, 8/24 astrocytomas, two of nine oligoastrocytomas and one of ten medulloblastomas. The 23 mutations consisted of 19 transitions, three transversions and one deletion of a single basepair resulting in a frameshift. Noteworthy are six identical C→T transitions in codon 273 in glial tumours, indicating that this codon represents a mutational hotspot in gliomas. Two G→A transitions each were found in codons 175 and 248 of four glioblastomas. One anaplastic oligoastrocytoma had mutations in codons 249 and 281. There was no association between any one of the WAF1/CIP1 polymorphisms and mutations in exons 5–8 of the p53 gene.

**Figure 1** Analysis of the WAF1/CIP1 gene in brain tumours. (**a**) SSCP patterns of the WAF1/CIP1 gene in brain tumours. The corresponding polymorphisms are listed in Table II. (**b**) Sequence analysis of the F2 allele in codon 39. The sequence of the prevalent F1 allele (C) is indicated.

**Table I** Polymorphisms in the WAF1/CIP1 gene

| *Allele* | *Allele frequency* | *Codon* | *Sequence* | *Amino acid* |
|---|---|---|---|---|
| A1 | 91% | 31 | AGC | Ser |
| A2 | 9% | 31 | AGA | Arg |
| B1 | 99.7% | 25 | GTG | Val |
| B2 | 0.3% | 25 | GGG | Gly |
| C1 | 99.7% | 64 | GCC | Ala |
| C2 | 0.3% | 64 | ACC | Thr |
| D1 | 99.7% | 32 | CGC | Arg |
| D2 | 0.3% | 32 | CTC | Leu |
| E1 | 99.7% | 14 | GGC | Gly |
| E2 | 0.3% | 14 | AGC | Ser |
| F1 | 99.7% | 39 | GCG | Ala |
| F2 | 0.3% | 39 | GTG | Val |

Codons affected, DNA sequence, amino acid exchange and allele frequencies are given for the six observed polymorphisms in the WAF1/CIP1 gene.

**Table II** WAF1/CIP1 haplotypes in 158 human brain tumours

| *Haplotype* | *Allele distribution* | *Frequency* |
|---|---|---|
| I | A1/A1,B1/B1,C1/C1,D1/D1,E1/E1,F1/F1 | 127/158 (80.4%) |
| II | A1/**A2**,B1/B1,C1/C1,D1/D1,E1/E1,F1/F1 | 25/158 (15.8%) |
| III | **A2/A2**,B1/B1,C1/C1,D1/D1,E1/E1,F1/F1 | 1/158 (0.63%) |
| IV | A1/A1,B1/**B2**,C1/C1,D1/D1,E1/E1,F1/F1 | 1/158 (0.63%) |
| V | A1/A1,B1/B1,C1/**C2**,D1/D1,E1/E1,F1/F1 | 1/158 (0.63%) |
| VI | A1/A1,B1/B1,C1/C1,D1/**D2**,E1/E1,F1/F1 | 1/158 (0.63%) |
| VII | A1/A1,B1/B1,C1/C1,D1/D1,E1/**E2**,F1/F1 | 1/158 (0.63%) |
| VIII | A1/A1,B1/B1,C1/C1,D1/D1,E1/E1,F1/**F2** | 1/158 (0.63%) |

Allele distribution and frequencies are listed for the eight observed haplotypes. The rare alleles characteristic for the haplotypes are given in boldface.

### Discussion

We report on a large series of brain tumours examined for mutations in the WAF1/CIP1 gene. No somatic mutations were found in 158 patients with brain tumours. Since we have only screened 87% of the coding sequence of the gene, we cannot exclude mutations in other regions, but an extensive study of the entire coding region of WAF1/CIP1 in non-cerebral tumours also failed to detect mutations (Shiohara *et al*., 1994). In addition, allelic loss of the WAF1/CIP1 region of chromosomal arm 6p was extremely rare. We therefore conclude that WAF1/CIP1 mutations are probably not involved in the formation of human brain tumours.

While no somatic mutations were detected in our study, we found a surprising number of different non-conservative polymorphisms. The most frequent polymorphism A1/A2 has been described in previous reports (Chedid *et al*., 1994; Shiohara *et al*., 1994). In our series the frequencies for A1/A2 were 0.91/0.09 resulting in 15.8% heterozygotes. This Ser/Arg polymorphism in codon 31 has been reported with a similar incidence (Chedid *et al*., 1994; Shiohara *et al*., 1994). We detected five additional polymorphisms (B–F) in the region of codons 14–64. All polymorphisms result in alterations of the amino acid sequence (see Table I). Two other studies have failed to detect any of the variants except for the common A1/A2 polymorphism (Chedid *et al*., 1994; Shiohara *et al*., 1994). All of these polymorphisms reside in an area of greater than 90% homology at the protein level with the murine homologue, which is thought to encode a DNA-binding zinc-finger domain (El-Deiry *et al*., 1993; Huppi *et al*., 1994). This observation raises the possibility that these polymorphisms encode functionally distinct proteins, but transfection studies have shown no difference in the tumour-suppressor abilities of the serine and arginine alleles (A1/A2) in a lung cancer cell line (Chedid *et al*., 1994). Interestingly, the A2 allele corresponds to the mouse sequence. The other, rarer polymorphisms, however, await functional characterisation. A recent report localised the Cdk kinase inhibiting activity to the N-terminal region of WAF1/CIP1 (Chen *et al*., 1995). All polymorphisms described in the present study reside in the proposed Cdk kinase inhibiting domain. Therefore, these polymorphisms may be useful in studying this particular function of WAF1/CIP1 in naturally occurring variants. Nonetheless, the occurrence of the B2 allele in a healthy individual argues against a pathogenic effect of this variant. Indeed, comparison of the allelic distribution in the tumour and control groups revealed a nearly identical distribution for the A1/A2 and B1/B2 alleles, and while the other, rarer polymorphisms were only seen in tumour patients, this association was not significant. Thus, although our study has demonstrated that somatic WAF1/CIP1 mutations do not occur in brain tumours and that none of the various WAF1/CIP1 polymorphisms predisposes to tumour formation, the study stresses the importance of combined analysis of paired tumour/constitutional DNA samples to avoid interpreting these multiple non-conservative polymorphisms as somatic mutations.

Mutations in the p53 gene were detected in 11/54 glioblastomas, 8/24 astrocytomas, two of nine oligoastrocytomas and one of ten medulloblastomas. These figures are similar to previous studies (Ohgaki *et al*., 1993; Louis, 1994). No association between p53 mutations and WAF1/CIP1 polymorphisms was noted. Therefore, while alterations of other components in the p53 cascade, such as MDM2 amplification may provide an alternative means for inactivating the p53 pathway (Reifenberger *et al*., 1993), WAF1/CIP1 does not appear to have such a role in human brain tumours.

**Acknowledgements**
We thank Dr Markus Nöthen from the Institute for Human Genetics, University of Bonn, Germany for providing 157 DNAs for our control group. We thank O Schmidt and H Klatt for their skilful technical assistance. This work was supported by the Deutsche Forschungsgemeinschaft (SFB 400), the Schäfersuolte Foundation, by NIH CA 57683 and by a centre grant from the University of Bonn and the state of Nordrhein Westfalen.

### References

BENDER B, WIESTLER OD AND VON DEIMLING A. (1994). A device for processing large acrylamide gels. *Biothechniques*, **16**, 204–206.

CHEDID M, MICHIELI P, LENGEL C, HUPPI K AND GIVOL D. (1994). A single nucleotide substitution at codon 31 (Ser/Arg) defines a polymorphism in a highly conserved region of the p53-inducible gene WAF1/CIP1. *Oncogene*, **9**, 3021–3024.

CHEN J, JACKSON PK, KIRSCHNER MW AND DUTTA A. (1995). Separate domains of p21 involved in the inhibition of Cdk kinase and PCNA. *Nature*, **374**, 386–388.

DI LEONARDO A, LINKE SP, CLARKIN K AND WAHL GM. (1994). DNA damage triggers a prolonged p53-dependent $G_1$ arrest and long-term induction of Cip1 in normal human fibroblasts. *Genes Dev*., **8**, 2540–2551.

EL-DEIRY WS, TOKINO T, VELCULESCCU VE, LEVY DB, PARSONS R, MERCER WE, KINZLER KW AND VOGELSTEIN B. (1993). WAF1, a potential mediator of p53 tumor suppression. *Cell*, **75**, 817–825.

EL-DEIRY WS, HARPER W, O'CONNOR PM, VELCULESCU VE, CANMAN CE, JACKMAN J, PIETENPOL JA, BURELL M, HILL DE, WANG Y, WIMAN KG, MERCER WE, KASTAN MB, KOHN KW, ELLEDGE SJ, KINZLER K AND VOGELSTEIN B. (1994). WAF1/CIP1 is induced in p53-mediated $G_1$ arrest and apoptosis. *Cancer Res*., **54**, 1169–1174.

HARPER JW, ADAMI GR, WEI N, KEYOMARSI K AND ELLEDGE J. (1993). The p21 cdk-interacting protein Cip1 is a potent inhibitor of G1 cyclin-dependent kinases. *Cell*, **75**, 805–816.

HUPPI K, SIWARSKI D, DOSIK J, MICHIELI P, CHEDID M, REED S, MOCK B, GIVOL D AND MUSHINSKY JF. (1994). Molecular cloning, sequencing, chromosomal localization and expression of mouse p21 (WAF). *Oncogene*, **9**, 3017–3020.

KLEIHUES P, BURGER PC AND SCHEITHAUER BW. (1993). *Histological Typing of Tumours of the Central Nervous System*. Springer: Berlin.

LANE DP. (1993). A death in the life of p53. *Nature*, **362**, 786–787.

LOUIS DN. (1994). The p53 gene and protein in human brain tumors. *J Neuropathol. Exp. Neurol.*, **53**, 11-21.

LOUIS DN, VON DEIMLING A AND SEIZINGER BR. (1992). A (CA)n dinucleotide repeat assay for evaluating loss of allelic heterozygosity in small and archival human brain tumor specimens. *Am. J. Pathol.*, **141**, 777-782.

OHGAKI H, EIBL RH, SCHWAB M, REICHEL MB, MARIANI L, GEHRING M, PETERSEN I, HÖLL T, WIESTLER OD AND KLEIHUES P. (1993). Mutations in the p53 tumor suppressor gene in neoplasms of the human nervous system. *Mol. Carcinogenesis*, **8**, 74-80.

REIFENBERGER G, LIU L, ICHIMURA K, SCHMIDT EE AND COLLINS VP. (1993). Amplification and overexpression of the MDM2 gene in a subset of human malignant gliomas without p53 mutations. *Cancer Res.*, **53**, 2736-2739.

RUBIO MP, VON DEIMLING A, YANDELL DW, WIESTLER OD, GUSELLA JF AND LOUIS DN. (1993). Accumulation of wild-type p53 protein in human astrocytomas. *Cancer Res.*, **53**, 3465-3467.

SAMBROOK J, FRITSCH EF AND MANIATIS T. (1989). *Molecular cloning*. Cold Spring Harbor Laboratory Press: Cold Spring Harbor, NY.

SHIOHARA M, EL-DEIRY WS, WADA M, NAKAMAKI T, TAKEUCHI S, YANG R, CHEN D-L, VOGELSTEIN B AND KOEFFLER P. (1994). Absence of WAF1 mutations in a variety of human malignancies. *Blood*, **84**, 3781-3784.

SMITH ML, CHEN IT, ZHAN Q, BAE I, CHEN C-Y, GILMER TM, KASTAN MB, O'CONNOR PM AND FORNACE JR, AJ. (1994). Interaction of the p53-regulated protein gadd45 with proliferating cell nuclear antigen. *Science*, **266**, 1376-1380.

VON DEIMLING A, EIBL RH, OHGAKI H, LOUIS DN, VON AMMON K, PETERSEN I, KLEIHUES P, CHUNG RY, WIESTLER OD AND SEIZINGER BR. (1992). p53 mutations are associated with 17p allelic loss in grade II and grade III astrocytoma. *Cancer Res.*, **52**, 2987-2990.

VON DEIMLING A, BENDER B, LOUIS DN AND WIESTLER OD. (1993). A rapid and non-radioactive PCR based assay for the detection of allelic loss in human gliomas. *Neuropathol. Appl. Neurobiol.*, **19**, 524-529.

XIONG Y, HANNON GJ, ZHANG H, CASSO D, KOBAYASHI R AND BEACH D. (1993). p21 is a universal inhibitor of cyclin kinases. *Nature*, **366**, 701-704.


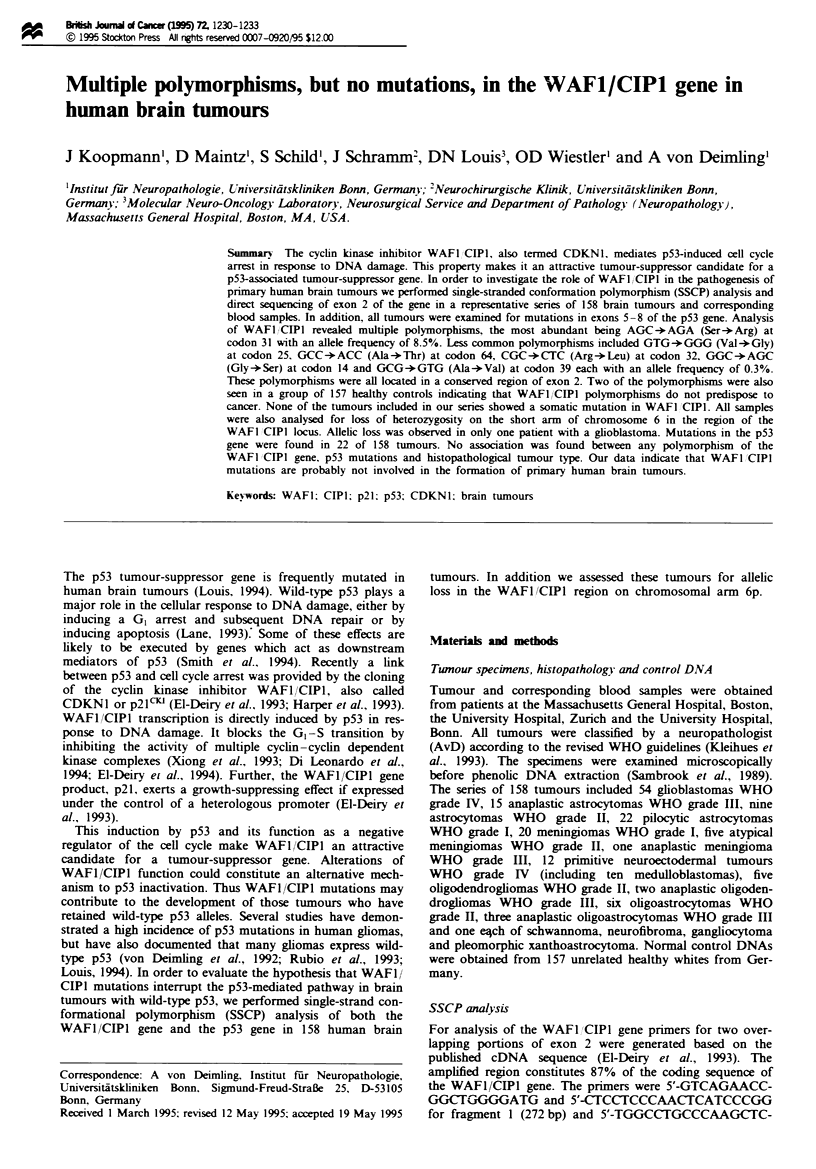

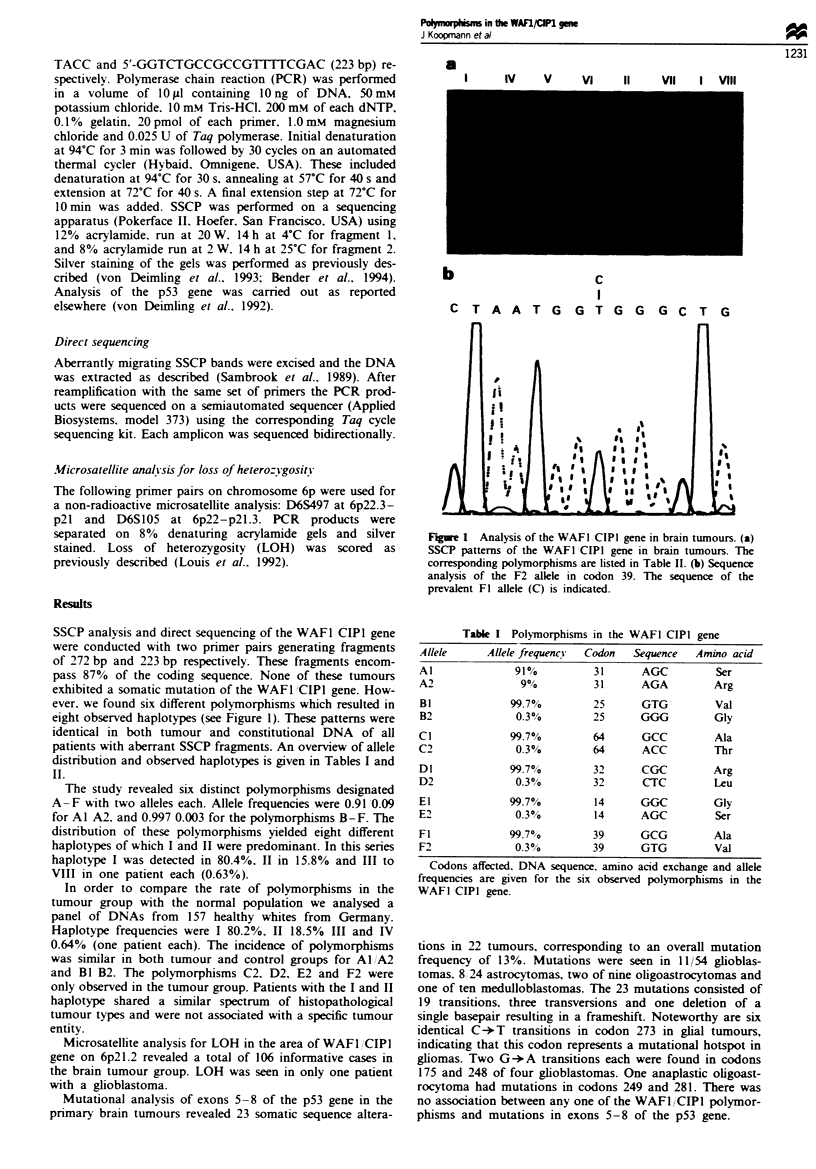

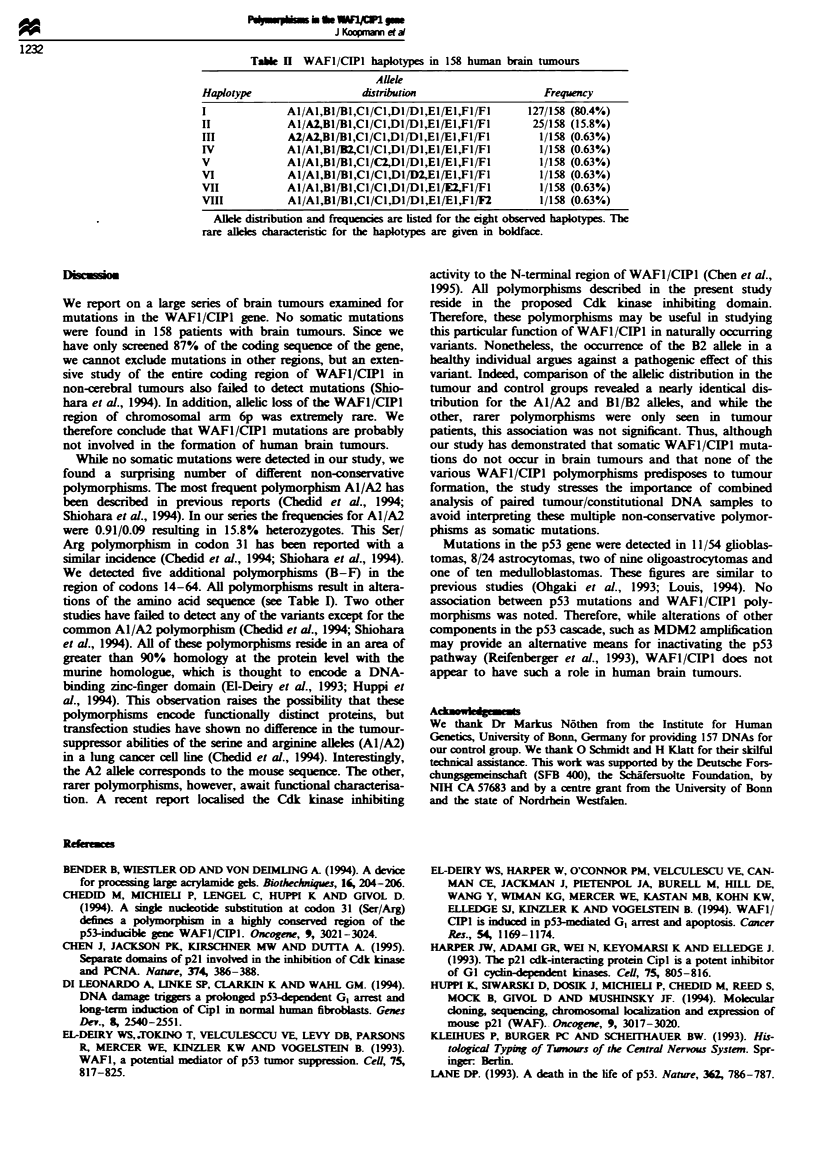

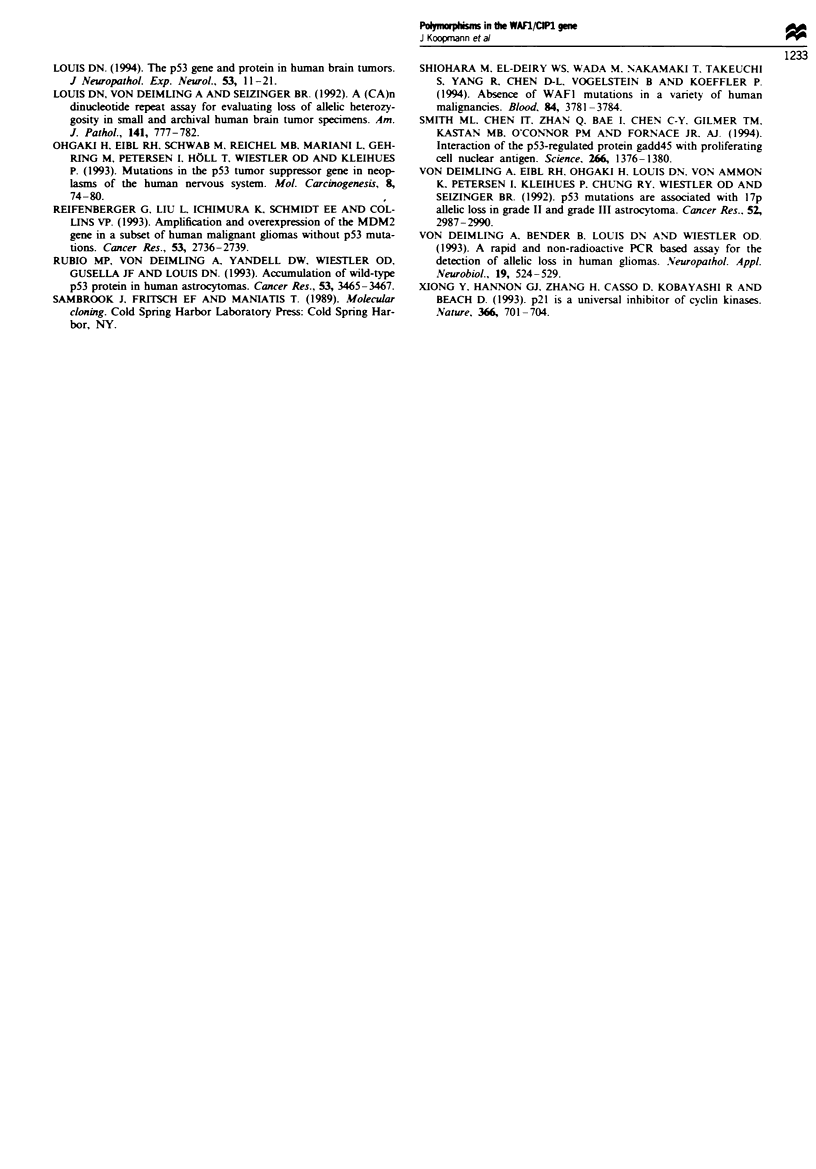


## References

[OCR_00351] Bender B., Wiestler O. D., von Deimling A. (1994). A device for processing large acrylamide gels.. Biotechniques.

[OCR_00356] Chedid M., Michieli P., Lengel C., Huppi K., Givol D. (1994). A single nucleotide substitution at codon 31 (Ser/Arg) defines a polymorphism in a highly conserved region of the p53-inducible gene WAF1/CIP1.. Oncogene.

[OCR_00362] Chen J., Jackson P. K., Kirschner M. W., Dutta A. (1995). Separate domains of p21 involved in the inhibition of Cdk kinase and PCNA.. Nature.

[OCR_00367] Di Leonardo A., Linke S. P., Clarkin K., Wahl G. M. (1994). DNA damage triggers a prolonged p53-dependent G1 arrest and long-term induction of Cip1 in normal human fibroblasts.. Genes Dev.

[OCR_00387] Harper J. W., Adami G. R., Wei N., Keyomarsi K., Elledge S. J. (1993). The p21 Cdk-interacting protein Cip1 is a potent inhibitor of G1 cyclin-dependent kinases.. Cell.

[OCR_00390] Huppi K., Siwarski D., Dosik J., Michieli P., Chedid M., Reed S., Mock B., Givol D., Mushinski J. F. (1994). Molecular cloning, sequencing, chromosomal localization and expression of mouse p21 (Waf1).. Oncogene.

[OCR_00404] Lane D. P. (1993). Cancer. A death in the life of p53.. Nature.

[OCR_00412] Louis D. N. (1994). The p53 gene and protein in human brain tumors.. J Neuropathol Exp Neurol.

[OCR_00416] Louis D. N., von Deimling A., Seizinger B. R. (1992). A (CA)n dinucleotide repeat assay for evaluating loss of allelic heterozygosity in small and archival human brain tumor specimens.. Am J Pathol.

[OCR_00423] Ohgaki H., Eibl R. H., Schwab M., Reichel M. B., Mariani L., Gehring M., Petersen I., Höll T., Wiestler O. D., Kleihues P. (1993). Mutations of the p53 tumor suppressor gene in neoplasms of the human nervous system.. Mol Carcinog.

[OCR_00430] Reifenberger G., Liu L., Ichimura K., Schmidt E. E., Collins V. P. (1993). Amplification and overexpression of the MDM2 gene in a subset of human malignant gliomas without p53 mutations.. Cancer Res.

[OCR_00436] Rubio M. P., von Deimling A., Yandell D. W., Wiestler O. D., Gusella J. F., Louis D. N. (1993). Accumulation of wild type p53 protein in human astrocytomas.. Cancer Res.

[OCR_00445] Shiohara M., el-Deiry W. S., Wada M., Nakamaki T., Takeuchi S., Yang R., Chen D. L., Vogelstein B., Koeffler H. P. (1994). Absence of WAF1 mutations in a variety of human malignancies.. Blood.

[OCR_00451] Smith M. L., Chen I. T., Zhan Q., Bae I., Chen C. Y., Gilmer T. M., Kastan M. B., O'Connor P. M., Fornace A. J. (1994). Interaction of the p53-regulated protein Gadd45 with proliferating cell nuclear antigen.. Science.

[OCR_00469] Xiong Y., Hannon G. J., Zhang H., Casso D., Kobayashi R., Beach D. (1993). p21 is a universal inhibitor of cyclin kinases.. Nature.

[OCR_00380] el-Deiry W. S., Harper J. W., O'Connor P. M., Velculescu V. E., Canman C. E., Jackman J., Pietenpol J. A., Burrell M., Hill D. E., Wang Y. (1994). WAF1/CIP1 is induced in p53-mediated G1 arrest and apoptosis.. Cancer Res.

[OCR_00371] el-Deiry W. S., Tokino T., Velculescu V. E., Levy D. B., Parsons R., Trent J. M., Lin D., Mercer W. E., Kinzler K. W., Vogelstein B. (1993). WAF1, a potential mediator of p53 tumor suppression.. Cell.

[OCR_00461] von Deimling A., Bender B., Louis D. N., Wiestler O. D. (1993). A rapid and non-radioactive PCR based assay for the detection of allelic loss in human gliomas.. Neuropathol Appl Neurobiol.

[OCR_00454] von Deimling A., Eibl R. H., Ohgaki H., Louis D. N., von Ammon K., Petersen I., Kleihues P., Chung R. Y., Wiestler O. D., Seizinger B. R. (1992). p53 mutations are associated with 17p allelic loss in grade II and grade III astrocytoma.. Cancer Res.

